# Multi-locus sequence typing of *Treponema pallidum* subsp. *pallidum* present in clinical samples from France: Infecting treponemes are genetically diverse and belong to 18 allelic profiles

**DOI:** 10.1371/journal.pone.0201068

**Published:** 2018-07-19

**Authors:** Petra Pospíšilová, Philippe Alain Grange, Linda Grillová, Lenka Mikalová, Pervenche Martinet, Michel Janier, Annie Vermersch, Nadjet Benhaddou, Pascal Del Giudice, Isabelle Alcaraz, François Truchetet, Nicolas Dupin, David Šmajs

**Affiliations:** 1 Department of Biology, Faculty of Medicine, Masaryk University, Brno, Czech Republic; 2 Institut Cochin U1016 Equipe Batteux, Laboratoire de Dermatologie–CNR Syphilis, Faculté de Médecine, Université Sorbonne Paris Descartes, Paris, France; 3 Service Prévention Santé Conseil Départemental des Bouches du Rhône, Marseille, France; 4 Centre des MST, Hôpital Saint-Louis, AP-HP, Paris, France; 5 Service de Dermatologie, Hôpital Jean Bernard, Valenciennes, France; 6 Service de Bactériologie, Groupe Hospitalier Paris Centre Cochin-Hôtel Dieu-Broca, Paris, France; 7 Service de Dermatologie-Infectiologie, Centre Hospitalier Inter régional, Fréjus, France; 8 Service Universitaire des Maladies Infectieuses et du Voyageur, Hôpital Dron, Tourcoing, France; 9 Service de Dermatologie, Hôpital Bel Air, Metz-Thionville, France; 10 Service de Dermatologie-Vénéréologie, Hôpital Cochin–Pavillon Tarnier, AP-HP, Paris, France; Universita degli Studi di Bologna, ITALY

## Abstract

*Treponema pallidum* subsp. *pallidum*, the causative agent of sexually transmitted syphilis, detected in clinical samples from France, was subjected to molecular typing using the recently developed Multilocus Sequence Typing system. The samples (n = 133) used in this study were collected from 2010–2016 from patients with diagnosed primary or secondary syphilis attending outpatient centers or hospitals in several locations in France. Altogether, 18 different allelic profiles were found among the fully typed samples (n = 112). There were five allelic variants identified for TP0136, 12 for TP0548, and eight for TP0705. Out of the identified alleles, one, seven, and three novel alleles were identified in TP0136, TP0548, and TP0705, respectively. Partial allelic profiles were obtained from 6 samples. The majority of samples (n = 110) belonged to the SS14-like cluster of TPA isolates while 7 clustered with Nichols-like isolates. Patients infected with Nichols-like samples were more often older (*p* = 0.041) and more often diagnosed with secondary syphilis (*p* = 0.033) compared to patients infected with SS14-like samples. In addition, macrolide resistance caused by the A2058G mutation was found to be associated with allelic profile 1.3.1 or with strains belonging to the 1.3.1 lineage (*p*<0.001). The genetic diversity among TPA strains infecting the European population was surprisingly high, which suggests that additional studies are needed to reveal the full genetic diversity of TPA pathogens infecting humans.

## Introduction

The causative agent of syphilis, *Treponema pallidum* subsp. *pallidum* (TPA; for review see [1]), infects more than 5.6 million people each year worldwide [2, 3] and has yet to be continuously cultivated under *in vitro* conditions. This fact complicates genetic and phenotypic characterization of treponemes including macrolide resistance testing [4]. Therefore, treponemal isolates propagated in rabbits were available for the research of this bacterium for many years and collections of these standard laboratory strains including Nichols and SS14 strains were used for TPA studies. The introduction of next generation sequencing resulted in an increasing number of complete and draft TPA genome sequences [5, 6] in the public databases and the accumulated genomic data provided excellent background for further studies. Genetic characterizations of the treponemal DNA in clinical samples represents a next step in the analyses of TPA strains present in the human population and could include also detection of macrolide resistance.

Molecular typing of TPA has been widely used since its introduction in 1998 [7]. The original TPA typing system introduced by Pillay and colleagues [7], designated as the CDC-typing scheme (CDCT), determined the number of 60 bp-long repeats in the *arp* gene (TP0433) together with restriction fragment length polymorphism of the *tpr*E (TP0313), *tpr*G (TP0317), and *tpr*J (TP0621) genes. Later, the CDCT was supplemented by including the number of repeats in the *rps*A gene (TP0279) (CDCT-*rps*A) [8] or by additional sequence analysis of the TP0548 locus, which was known as the Enhanced CDC typing system (ECDCT) [9]. In 2006, a sequencing-based molecular typing (SBMT) system that determined the sequences of TP0136, TP0548, and 23S rDNA was introduced [10–13]. The CDC and ECDC typing systems (reviewed in [14–16]) have revealed several associations between TPA types and specific patient parameters including the prevalence of the 14d/f type in neurosyphilis [9], prevalence of the 14d/g type in macrolide resistant samples [17], and an increased frequency of the 14i/a type in serofast patients [18]. SBMT of TPA-containing human samples revealed differences in the geographical distribution of syphilis-causing strains [19], showed changing temporal patterns in the same geographical area [20], showed an association between certain TPA genotypes with mutations causing macrolide resistance [19–21], and showed associations between certain TPA genotypes and groups of patients including men having sex with men (MSM) [20]. The long-term use of (E)CDC typing system revealed some of the weaknesses of this systems including absence of controls indicating, during multiplex amplification of the genes *tpr*E, G, and J, equal amplification efficiency of these genes. Moreover, detected CDC subtypes did not correlate with phylogeny of TPA strains and isolates [4, 20, 22]. Furthermore, instability of the typing loci was described [23] and low success rate of fully typed samples was often achieved ([19, 24–26]. Although the number of fully typed samples is influenced by the quality of original clinical specimens, storage conditions and subsequent DNA isolation and PCR amplification procedures, the exclusion of *arp* repetitive sequence amplifications could result in better success rate.

In syphilis patients with negative serology, TPA molecular typing helps, in many cases, to determine a syphilis diagnosis [27]. Moreover, TPA molecular typing has already revealed misdiagnosed cases of syphilis when the infections were, in fact, caused by *Treponema pallidum* subsp. *endemicum* [28–31].

Recently, the SBMT system was supplemented with additional sequencing of TP0705 and designated as the Multilocus Sequence Typing system (MLST) [21]. The change increased genotype resolution power of TPA SS14-like clinical samples [32–34], which are the most common samples among clinical samples in Europe and USA [35]. Moreover, the stability of these typing loci was demonstrated [11]. Since 2 of the targets (TP0136 and TP0548) code for outer membrane proteins [36, 37], that are likely under host immune pressure, these loci represent excellent targets to evaluate strain diversity.

In this study, using the new MLST system, we mapped 133 clinical samples collected from 2010–2016 in several French cities including Marseille and Paris. Among these samples we identified 18 different TPA allelic profiles, of which 10 represent new, undescribed allelic profiles.

## Material and methods

### Collection of clinical material

Samples were collected from patients with primary or secondary syphilis coming from different locations in France (Aix-en-Provence (n = 8), Fréjus (n = 1), Marseille (n = 23), Metz-Thionville (n = 10), Nancy (n = 6), Paris (n = 79), Tourcoing (n = 2), Valenciennes (n = 3), and Martinique (1). Samples were collected from 2010–2016. Clinical data including patient age, gender, sexual orientation, HIV status, type of clinical material, syphilis serology results, and syphilis stage were collected when possible. HIV status was determined based on clinical data. Genital, anal, oral, and cutaneous ulcer swabs were collected and immediately examined using dark-field microscopy (DFM) and sent to the Centre National de Référence de la syphilis (CNR syphilis) for molecular analysis. Samples that tested positive during diagnostic nested PCR (nPCR) targeting of TP0574 [38] were then eligible for MLST.

### Diagnosis of syphilis

The diagnosis of clinically active early syphilis (primary or secondary) was based on criteria established by the Center for Disease Control (CDC) in 1997 [39]. All patients classified as having probable or confirmed syphilis were considered to have syphilis for the purposes of this study. Primary syphilis was confirmed when the patient presented with one or more chancres associated with the detection of *T*. *pallidum* using DFM. The diagnosis was considered probable when the patient presented with one or more chancres or ulcers consistent with a diagnosis of primary syphilis and a positive serologic test. Secondary syphilis was confirmed in patients presenting with cutaneous and/or mucosal lesions, localized or diffuse, with or without regional lymphadenopathy, and associated with direct detection of *T*. *pallidum* using DFM. A diagnosis of secondary syphilis was considered probable for patients presenting with the clinical criteria described above and having positive non-treponemal and treponemal serological tests. The diagnosis of primary/secondary syphilis was confirmed in patient presenting one or more chancres or ulcers consistent with the diagnosis of primary syphilis and also cutaneous and/or mucosal lesions, localised or diffusely present on body site, with or without regional lymphadenophaty and associated with a positive serology.

### Serological tests

Serum samples from all patients included in this study were tested using the RPR test (Bio-Rad, Marnes la Coquette, France) and Architect Syphilis TP assay (Abbott Laboratories, Abbott Park, Green Oaks, IL, USA) according to the manufacturers’ instructions.

HIV-1 and HIV-2 antigens and antibodies detection in serum was performed in routine by an automated ELISA assay (Genscreen ULTRA HIV Ag-Ab®; Bio-Rad, Marne-la-Coquette, France) according to the manufacturers’ instructions. Positive detection of antibodies was confirmed by HIV-1 and HIV-2 immunoblottings (NEW Lav-Blot I and New Lav-Blot II; Bio-Rad, Marne-la Coquette, France).

### Isolation of DNA

Lesion exudates, from mucosal ulceration or erosion, were collected with a swab that was immediately placed in 1 ml of sterile PBS and stored at −20°C until DNA extraction processing. DNA was extracted from swab exudates using a Nucleospin® Blood kit (Macherey-Nagel Eurl, Hoerd, France), according to the manufacturer’s instructions. Briefly, 200 μl of sample were combined with the same volume of lysis buffer containing guanidine hydrochloride, tween 20, and proteinase K at 1.4 mg/ml and lysed for 30 min at 70°C. From cutaneous biopsy and intracardiac blood, 50 μg and 200 μl, respectively, were lysed with proteinase K at 56°C for 18 h under shaking and DNA was extracted using the NucleoSpin Tissue kit protocol (Macherey-Nagel Eurl, Hoerd, France). All DNA samples were stored at 4°C, for no more than two days, before testing, thus avoiding the need for repeated freeze/thaw cycles. Long term storage was done at −20°C.

### Detection of treponemal DNA and molecular typing

A set of 133 samples, which were positive after nested PCR targeting of TP0574 [38], were subjected to molecular typing of the TPA DNA present in the isolate. Typing consisted of amplification and sequencing of 3 chromosomal loci (partial sequences of TP0136, TP0548, and TP0705) and amplification and analysis of macrolide resistance loci (positions 2058 and 2059 in both 23S rRNA genes). Amplicons were obtained as previously described (Grillová et al., 2018) using nested PCR. The PCR mixture in the first step contained 2 μl of a 2.5 mM deoxynucleotide triphosphate (dNTP) mixture, 5 μl of 5x PS GXL buffer, 1 μl of each primer (10 pmol/μl), 0.1 μl of PrimeSTAR GXL polymerase (Takara Bio Europe, France), and 1 μl of DNA. PCR-grade water was added to yield a final volume of 25 μl. A DNA sample of *T*. *pallidum* subsp. *pallidum* strain Nichols (5 pg/μl) served as a positive control. As the first step, PCR amplification of all tested loci was performed under the following cycling conditions: 94°C (1 min); 98°C (10 s), 68°C (15 s) touch down (−1.0°C per cycle), and 68°C (1 min, 45 s) for 8 cycles; 98°C (10 s), 61°C (15 s), and 68°C (1 min, 45 s) for 35 cycles, with final extension at 68°C (7 min). In step 2, the PCR mixture contained 0.5 μl of a 10 mM deoxynucleotide triphosphate (dNTP) mixture, 2.5 μl of ThermoPol Reaction buffer, 0.25 μl of each primer (100 pmol/μl), 0.05 μl of *Taq* polymerase (5,000 U/ml; New England BioLabs, Ipswich, MA, USA), and 1 μl of the step one PCR product. PCR-grade water was added to yield a final volume of 25 μl. The second PCR amplification of all tested loci was performed under the following cycling conditions: 94°C (1 min); 94°C (30 s), 48°C (30 s), and 72°C (1 min, 15 s) for 40 cycles; and 72°C (7 min). A list of all the primers used can be found in S1 Table [21]. PCR products were purified using a QIAquick PCR Purification Kit (Qiagen, Hilden, Germany), according to the manufacturer's instructions, and sequenced using the dideoxy-terminator sequencing approach (GATC-Biotech AG, Constance, Germany). Sequence analyses were performed using Lasergene software (DNASTAR v. 7.1.0.; DNASTAR, Madison, WI, USA). Sequences representing new allelic variants in the described typing loci were deposited in the GenBank under accession numbers: MH105906 (TP0136), MH105907-13 (TP0548) and MH105914-16 (TP0705). Sequences from both amplified copies of the 23S rRNA genes were only evaluated at positions corresponding to positions 2058 and 2059 in the 23S rRNA gene of *Escherichia coli* (accession no. V00331), where A→G mutations have been shown to cause macrolide resistance [12, 40, 41].

### Phylogenetic analyses

Phylogenetic trees were generated with MEGA 7 using the bootstrapping Maximum-likelihood algorithm and the Tamura Nei model [42], and with Network software using the Median Joining algorithm [43]. Sequence Matrix 1.8 software was used for sequence concatenations [44].

### Statistical methods

Clinical characteristic correlations with typing results were tested using the Fisher’s exact test and statistical significance was set at p < 0.05 (two-sided test). Statistical analyses were performed using STATISTICA software v.12 (StatSoft, Tulsa, OK, USA).

### Ethics statement

The study was approved by the Institutional Review Board of the Comité de Protection des Personnes d’Ile de France 3 (S.C.3005) and was conducted according to the Declaration of Helsinki Principles. Epidemiological data were collected anonymously during consultations with physicians and using a specifically designed form as part of the National Syphilis Surveillance Infection program in France.

## Results

### Clinical characteristics of patients

The samples (n = 133) used in this study were collected from 2010–2016, from patients diagnosed with primary or secondary syphilis attending outpatient centers or hospitals in several different locations in France (see Material and Methods section for details). The majority of samples came from Paris and Marseille (n = 102; 76.7%). Clinical characteristics of patients in the study are shown in Table 1. Primary or secondary syphilis was diagnosed as described in the Material and Methods section. Only PCR-positive samples (targeting the TP0574 locus) were analyzed in this study.

**Table 1:** Clinical characteristics of patients with diagnosed syphilis.

*Characteristic*	Patients (n = 133)
Mean age, yr (range)	37 (0–71)
Sex, n (%)	Male: 129 (97); Female: 4 (3)
MSM^1^, n (%)	80/90 (88.9)
HIV infection, n (%)^2^	27/87 (31)
***Type of clinical material***	
Genital lesion	73/133 (54.9)
Anal lesion	14/133 (10.5)
Buccal lesion	32/133 (24.1)
Cutaneous lesion	12/133 (9)
Cutaneous biopsy	1/133 (0.75)
Intracardiac blood of 28-week-old fetus	1/133 (0.75)
***Stage of syphilis*, *n (%)***^***3***^	
Primary	61/99 (61.6)
Primary/Secondary	3/99 (3)
Secondary	35/99 (35.4)

^1^MSM, men having sex with men. No sexual orientation information (n = 43 samples).

^2^HIV status was determined based on clinical data. No HIV status information (n = 46 samples).

^3^No disease stage information (n = 34 samples).

### Amplification efficiency

The highest amplification efficiency was found for 23S rDNA (n = 118, 88.7%), followed by TP0705 (n = 117, 88%), TP0136 (n = 116, 87.2%), and TP0548 (n = 114, 85.7%). Complete allelic profiles, i.e., positive amplification at all tested typing loci (TP0136, TP0548, and TP0705) were obtained for 112 samples (84.2%).

### Typing of clinical samples based on TP0136, TP0548, and TP0705

**Fig 1:**
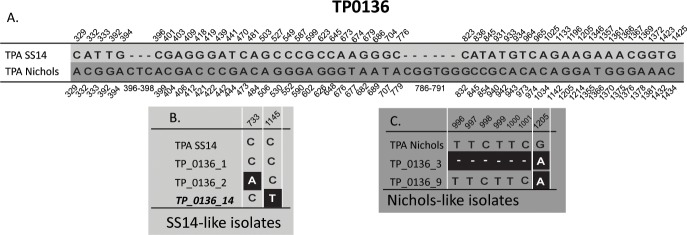
Alignment of the different TP0136 allelic variants identified in this study. Only positions containing nucleotide variants are shown. Only allelic variants identified in this study are shown; for all allelic variants identified so far, see S1 Appendix. A. Nucleotide differences between reference strain TPA SS14 and TPA Nichols in the region characterized using MLST (at coordinates 264–1469 according to the TPANIC_0136; CP004010.2). Coordinates shown above correspond to TPASS_0136 (TPA SS14; CP004011.1) and coordinates shown below correspond to TPANIC_0136 (TPA Nichols; CP004010.2). B. Nucleotide differences in SS14-like allelic variants. Coordinates correspond to TPASS_0136 (TPA SS14; CP004011.1). Allelic variants, which were not described previously, are shown in bold italics. **C**. Nucleotide differences in Nichols-like allelic variants. Coordinates correspond to TPANIC_0136 (TPA Nichols; CP004010.2).

**Fig 2:**
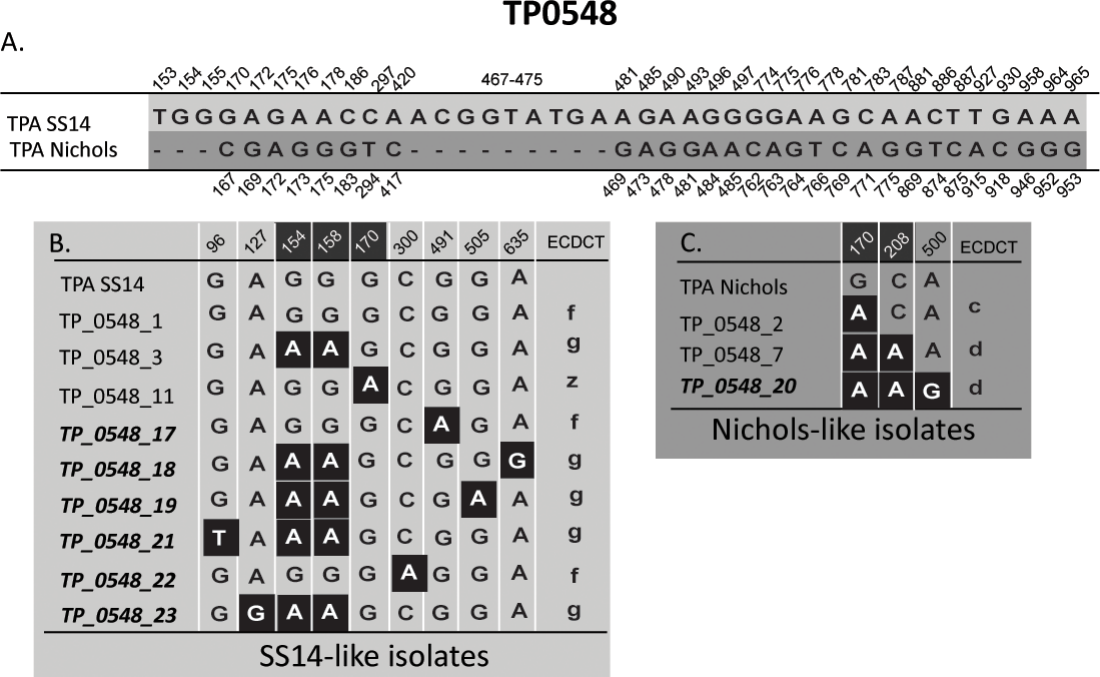
Alignment of different TP0548 allelic variants identified in this study. Only positions containing nucleotide v**ar**iants are shown. Only allelic variants identified in this study are shown; for a complete set of allelic variants see S1 Appendix. Coordinates shown in white on the dark background correspond to regions determined using ECDCT (Marra et al. 2010). All new allelic variants identified in this study included sequence changes outside the region characterized by ECDCT. A. Nucleotide differences between reference strain TPA SS14 and TPA Nichols in the region characterized using MLST (at coordinates 16–1080 of TPANIC_0548; CP004010.2). Coordinates shown above correspond to TPASS_0548 (TPA SS14; CP004011.1) and coordinates shown below correspond to TPANIC_0548 (TPA Nichols; CP004010.2). B. Nucleotide differences in SS14-like allelic variants. Coordinates correspond to TPASS_0548 (TPA SS14; CP004011.1). Allelic variants, which were not described previously, are shown in bold italics. The translation to ECDCT subtypes is shown in the last column. **C**. Nucleotide differences in Nichols-like allelic variants. Coordinates correspond to TPANIC_0548 (TPA Nichols; CP004010.2). Allelic variants, which were not described previously are shown in bold italics. The translation to ECDCT subtypes is shown in the last column.

**Fig 3:**
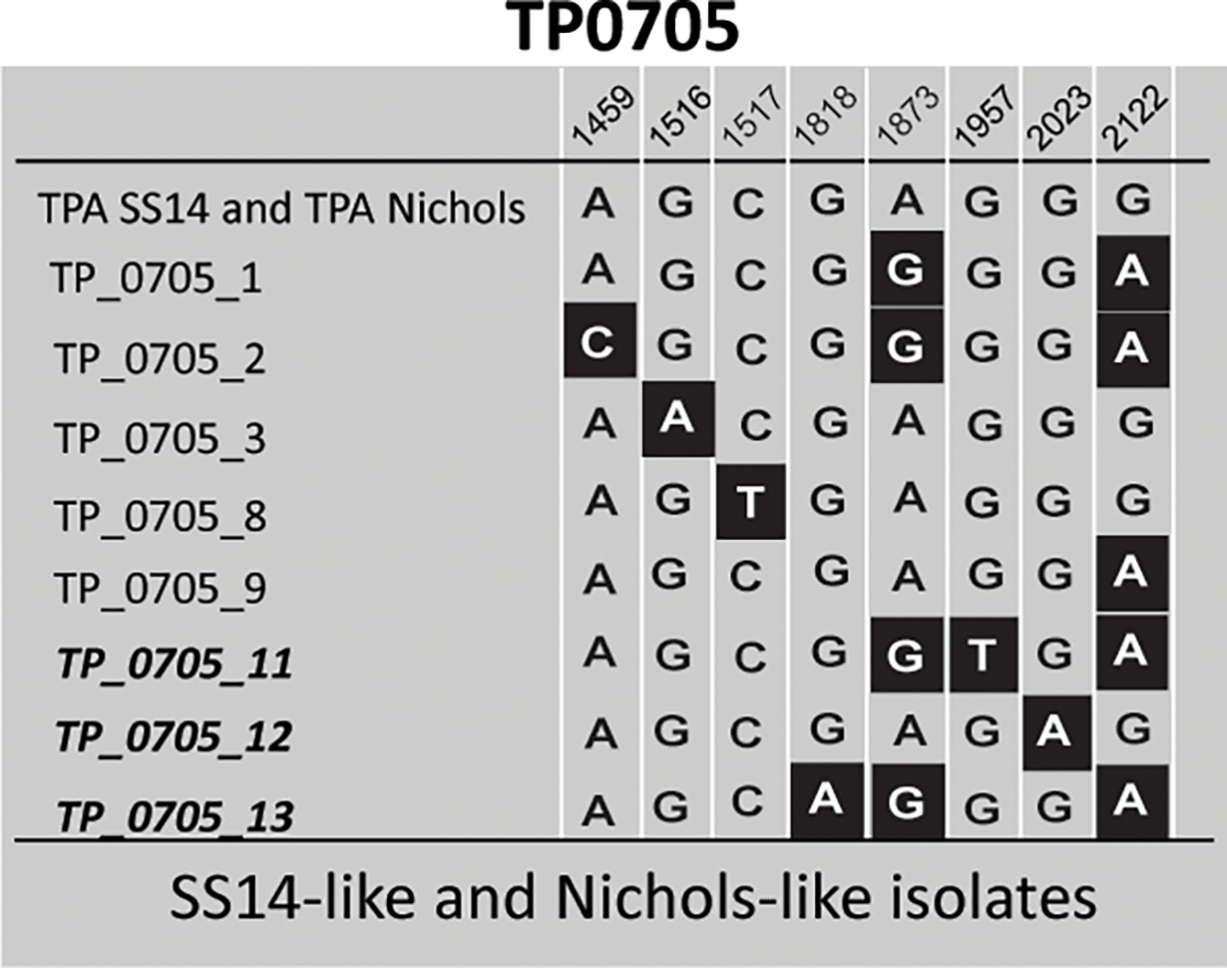
Alignment of different TP0705 allelic variants identified in this study. Coordinates correspond to TPASS_0705 and TPANIC_0705 (TPA SS14, CP004011.1; TPA Nichols, CP004010.2). Only positions containing nucleotide variants are shown (at coordinates 1368–2171 of TPANIC_0705; CP004010.2). Only allelic variants identified in this study are shown; for other allelic variants see S1 Appendix. Allelic variants, which were not described previously, are shown in bold italics. Note that the TP0705 gene is encoded by the complementary DNA strand relative to TP0136 and TP0548.

Altogether, 18 different allelic profiles were found among the tested samples (Table 2). There were five allelic variants identified for TP0136, 12 for TP0548, and eight for TP0705 (Figs 1–3). Out of the identified alleles, one, seven and three novel alleles were identified in TP0136, TP0548, and TP0705, respectively. Partial allelic profiles were obtained for 6 samples. No new allelic variants were identified among the partially typed samples.

**Table 2:** MLST allelic profiles of typed samples.

Allelic profile^1^	Typing	TP_0136 allelic variant^2^	TP_0548 allelic variant^2^	TP_0705allelic variant^2^	23S rDNA^3^	Genetic group^4^	Frequency^5^
**1.1.1**	Complete	1	1	1	S (6)/R8 (10)	SS14-like	16
**1.1.9**	Complete	1	1	9	S	SS14-like	1
**1.1.11**	Complete	1	1	11	R8	SS14-like	1
**1.1.13**	Complete	1	1	13	R8	SS14-like	1
**1.17.9**	Complete	1	17	9	R8	SS14-like	2
**2.1.2**	Complete	2	1	2	S	SS14-like	1
**1.3.1**	Complete	1	3	1	S (1)/R8 (68)/UN (1)	SS14-like	70
**1.18.1**	Complete	1	18	1	R8	SS14-like	1
**1.19.1**	Complete	1	19	1	R8	SS14-like	1
**1.21.1**	Complete	1	21	1	R8	SS14-like	1
**1.23.1**	Complete	1	23	1	R8	SS14-like	1
**14.3.1**	Complete	14	3	1	R8	SS14-like	1
**1.1.8**	Complete	1	1	8	S (1)/R8 (4)	SS14-like	5
**1.11.8**	Complete	1	11	8	R8	SS14-like	2
**1.22.12**	Complete	1	22	12	S	SS14-like	1
**9.7.3**	Complete	9	7	3	S (1)/R8 (4)	Nichols-like	5
**9.20.3**	Complete	9	20	3	S	Nichols-like	1
**3.2.3**	Complete	3	2	3	R8	Nichols-like	1
**1.X.1**	Partial	1	NA^6^	1	S (1)/R8 (1)	SS14-like	2
**1.3.X**	Partial	1	3	NA^6^	R8	SS14-like	1
**1.X.9**	Partial	1	NA^6^	9	R8	SS14-like	1
**X.17.9**	Partial	NA^6^	17	9	R8	SS14-like	1
**X.X.3**	Partial	NA^6^	NA^6^	3	S	unknown	1

^1^Allelic profiles are based on a three-number code: the first number corresponds to the allelic variant of the TP0136 locus, the second corresponds to the allelic variant of the TP0548 locus and the third corresponds to the allelic variant of the TP0705 locus [21].

^2^Single nucleotide variations (SNVs) that determine the allelic variants are shown in the Figs 1–3.

^3^S –no mutation in 23S rDNA (sensitive), R8 –A2058G mutation in 23S rDNA (resistance), UN–unknown. In cases where sensitive and resistant cases were found for one profile the frequency is given in parenthesis. Sequences of both copies of rDNA locus were determined.

^4^According to Nechvátal and colleagues [33].

^5^Frequency refers to the number of samples in which the corresponding allelic profile was found.

^6^NA, not available.

The majority of samples (n = 110) belonged to the SS14-like cluster of TPA samples while 7 were clustered with Nichols-like samples. A set of 16 samples remained unclassified, out of which 14 were not typeable at any of the tested loci, one was typeable at the TP0705 and 23S rDNA loci and for the remaining one, only the 23S rDNA locus sequence was obtained.

**Fig 4:**
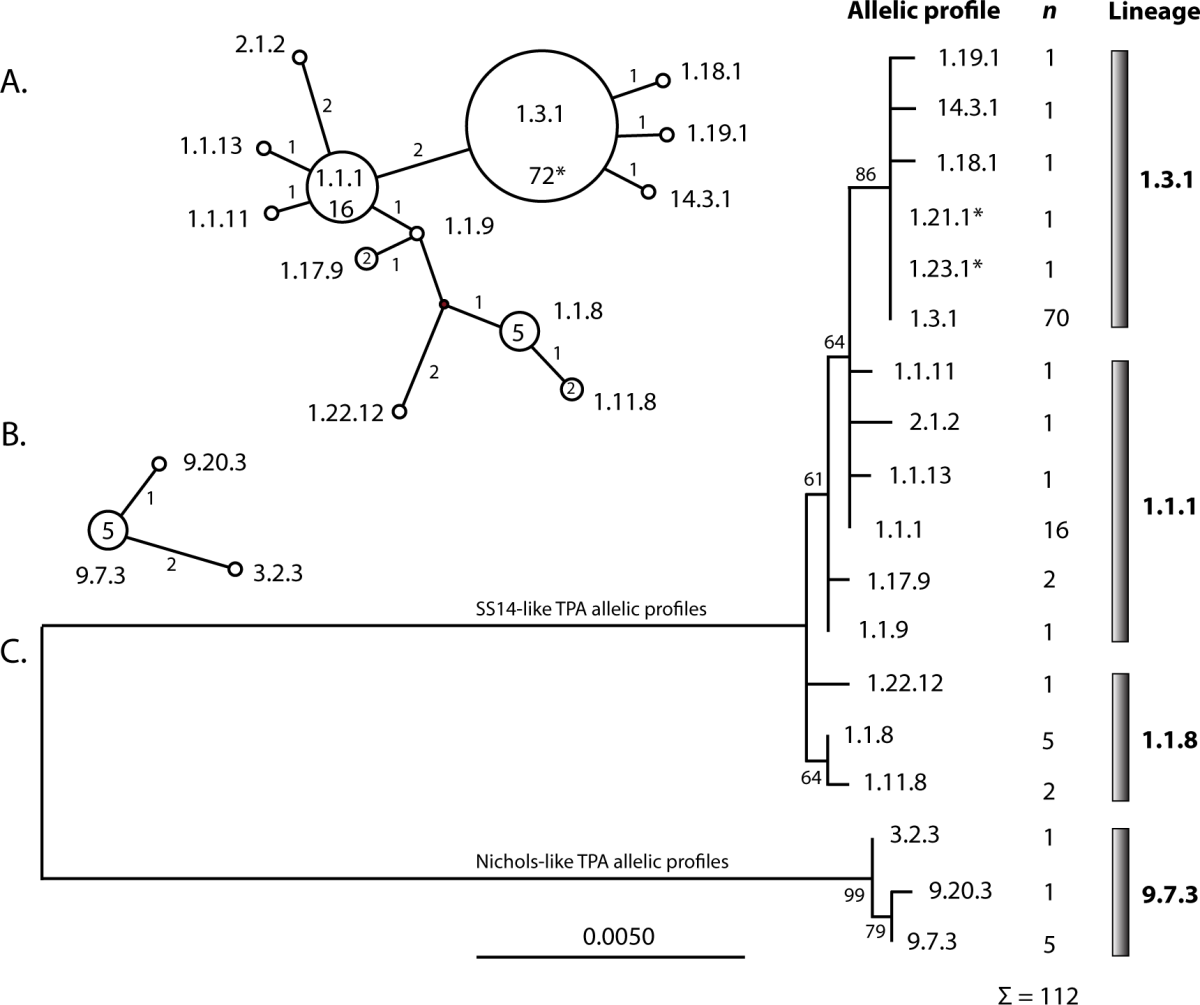
Phylogeny of allelic profiles of fully typed samples identified in this study. A. The Median joining network tree of concatenated sequences of TP0136, TP0548, and TP0705 of the SS14-like clinical samples (2526 nt in length in TPA SS14). The analyzed coordinates correspond to 336–1385 of TPANIC_0136, 150–951 of TPANIC_0548, and 1453–2126 of TPANIC_0705 (TPA Nichols; CP004010.2). Every circle represents a different allelic profile and specific allelic profiles are shown within or near the circles. The number of mutations are shown next to the branch lines. The inferred haplotype is shown as a black (connecting) circle. The size of the circle represents the number of identified samples and the corresponding numbers of samples are shown inside the circles (if greater than one). B.The Median joining network tree of concatenated sequences of TP0136, TP0548, and TP0705 of the Nichols-like clinical samples. Every circle represents a different allelic profile and specific allelic profiles are shown near the circles. The number of mutations are shown next to the branch lines. A contiguous indel was considered to be a single event. The size of the circle represents the number of identified samples and the corresponding number of samples are shown inside the circles (if greater than one). C. A phylogenetic tree constructed from concatenated sequences TP0136, TP0548, and TP0705. *Sequences of samples representing allelic profiles 1.21.1 and 1.23.1 also comprise nucleotide differences in positions 96 and 127 in TP0548 that were not used for the construction of the tree, respectively. Since sequences from additional samples were not obtained in this region, both allelic profiles 1.21.1 and 1.23.1 appear to be identical to allelic profile 1.3.1 (panel A. and C).

**Fig 5:**
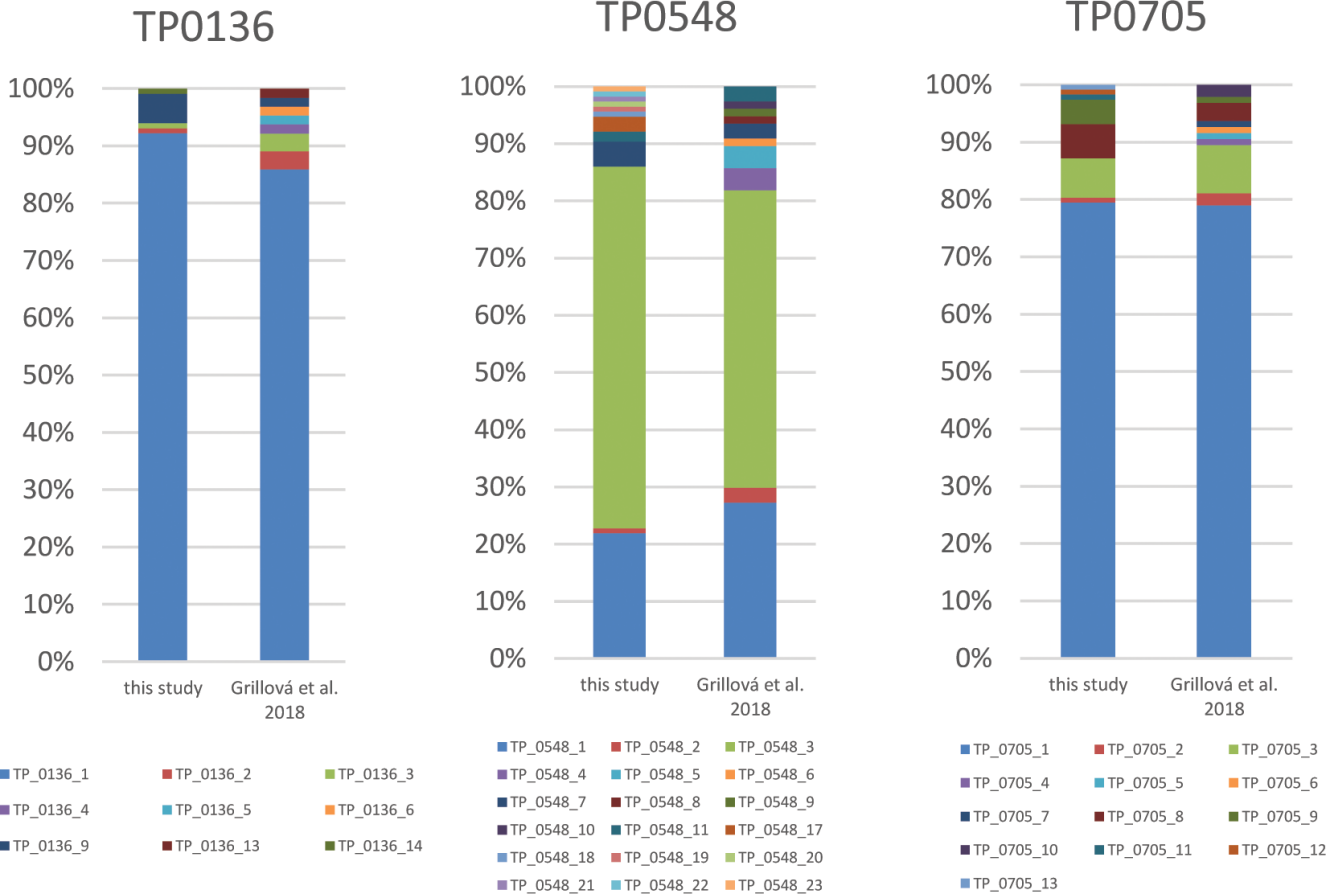
Comparison of allelic frequencies at individual typing loci identified in this study compared to results obtained by Grillová and coleagues [21].

The detected allelic profiles showed different frequencies (Table 2, Fig 4) with the most frequent profiles including 1.3.1 (n = 70), 1.1.1 (n = 16), 1.1.8 (n = 5), and 9.7.3 (n = 5). However, 12 different complete allelic profiles were found only in one patient (i.e. one allelic profile in one patient only, see profiles in Table 2 with frequency value 1). The spectra of allelic variants identified in this study were compared to data obtained from Switzerland and France [21] (Fig 5). One or two allelic profiles prevailed, to a similar extent, at all tested loci in both studied populations. On the other hand, allelic profiles with a low prevalence (i.e., those found in one or only a few patients) differed between both studies (Fig 5).

### Prevalence of macrolide resistance-causing mutations in clinical samples

Out of 133 samples examined in this study, the 23S rDNA was amplified and sequenced in 118 samples from 118 patients (88.7%). Altogether, 102 samples (86.4%) harbored the A2058G mutation in both *rrn* operons. None of the examined samples contained the A2059G mutation. Interestingly, four different MLST profiles (1.3.1; 1.1.1; 1.1.8, and 9.7.3) were found to contain samples harboring both wildtype and A2058G-containing 23S rDNA sequences. Mutations causing macrolide resistance were detected in both SS14-like and Nichols-like clinical samples (88.2% and 71.4%, respectively).

### Association of TPA genetic variants and clinical characteristics of patients

All available patient characteristics including geographical origin, age, gender, MSM status, HIV infection, serological test results, clinical material type, and syphilis stage were examined with regard to PCR positivity, macrolide resistance mutations, allelic variants and profiles, and genetic groups (SS14-like and Nichols-like). Associations between allelic profiles belonging to SS14-like or Nichols-like clades were found with respect to patient age and disease stage. Patients infected with Nichols-like TPA were more often older than 40 years (*p* = 0.041) and more often diagnosed with secondary syphilis (*p* = 0.033) compared to patients infected with SS14-like TPA. In addition, macrolide resistance (the A2058G mutation in both *rrn* operons) was found to be associated with allelic profile 1.3.1 or with samples belonging to the 1.3.1 lineage (*p* < 0.001) (Table 3, Fig 4).

**Table 3:** Associations of allelic profiles (or allelic profile lineage^‡^) and mutations causing macrolide resistance.

Allelic profile (Allelic profile lineage)	Macrolide sensitive	Macrolide resistant (A2058G)	*p* value
1.3.1*	1	68	
1.1.1	6	10	*p* = 0.0001
1.3.1 lineage*	1	73	
1.1.1 lineage	8	14	*p* = 0.000019
1.3.1 lineage*	1	73	
other samples	12	25	*p* = 0.0000047

^‡^allelic profile lineage is a group of allelic profiles related to each other based on sequence similarity (as illustrated in Fig 4, panel C). The names of the lineages were derived from the most prevalent allelic profile in the lineage.

*in 1 sample of allelic profile 1.3.1, the 23S rDNA locus PCR product was unsuccessfully amplified.

## Discussion

In this study, we have identified 18 different allelic profiles among 112 fully typed samples collected from patients attending outpatient centers or hospitals in France from 2010–2016. A previous study analyzing 120 samples from patients living in France and Switzerland (using the same typing technique [21]), revealed 23 different allelic profiles among 97 typeable TPA samples. Even though the samples were collected during a similar time period and came from partially overlapping geographic areas, only five allelic profiles were detected in both studies while 18 allelic profiles were found to be unique for the previous study [21] and 13 allelic profiles were unique for this study. Moreover, only the most abundant allelic profiles (1.3.1 and 1.1.1) overlapped. Similarly, when samples from this and the previous study [21] were analyzed with respect to their origin in France and Switzerland, only four overlapping allelic profiles were found, suggesting that the TPA strains infecting humans differ between subpopulations of patients.

Allelic profile 1.3.1, which was found to be the most frequently detected TPA allelic profile among French samples in this study, corresponds to the SU2 genotype based on SBMT [11] and was the most frequent genotype in the Czech Republic and Belgium [20, 45]. At the same time, the SU2 genotype corresponds to the “g” ECDCT_TP0548 subtype based on ECDCT [9] and this TPA subtype was found to be predominant in the US and in several European countries, as well as Australia [17, 20, 46–50]. On the other hand, several other countries including Argentina [19], China [18, 51–55], Taiwan [56], and Russia [57] have a higher prevalence of the “f” ECDCT_TP0548 subtype (allelic variant TP_0548_1) compared to the prevalence of the “g” ECDCT_TP0548 subtype, suggesting that there are important geographical differences in the most prominent TPA genotypes.

All allelic variants of TP0548, which were newly found in this study, had single nucleotide changes in the region outside the TP0548 gene fragment (0.08 kb in length), which is the target of ECDC typing [9]; this finding suggests that a larger portion of the TP0548 locus should be used for molecular typing compared to the more limited region currently analyzed using ECDCT [9]. Interestingly, ECDCT TP0548 subtypes d, f and g were further differentiated using longer PCR products of the TP0548 locus (Fig 2). In fact, there were 19 different ECDCT subtypes of TP0548 identified among 1904 samples tested in several studies (data taken from [5, 18, 48, 50, 53, 56, 58]. In comparison, among the 191 samples characterized by MLST in this and the previous study [21], 18 different allelic variants of TP0548 were found. Therefore, discoveries of new allelic TP0548 variants could be expected in the future studies.

Analysis of allelic variants detected in this study revealed that out of 23 single nucleotide replacements identified for all analyzed loci (TP0136, TP0548, and TP0705) and compared either to TPA Nichols or to TPA SS14 genomes, 21 (91.3%) resulted in amino acid replacements in the corresponding proteins (S2 Table). Similar results were also found in the work of Grillová and colleagues [21]. These findings suggest that the typing loci used for analysis evolve under positive selection during human TPA infection and represent molecular adaptations of TPA. At the same time, possible positive selection of typing loci opens the question of genetic stability of such regions. The typing stability of the TP0136 and TP0548 loci was analyzed in the work of Flasarová and colleagues [11] where epidemiologically related patients showed identical sequences for up to 31 days. An analysis of the rabbit-propagated TPA DAL-1 [59] strain showed that the TP0136, TP0548, and TP0705 loci remained stable for at least 142 days [21]. Moreover, the “SSS” genotype [11] was detected in the Czech Republic in all analyzed years from 2004 until 2013 [20] suggesting that, at least in some of TPA strains, the TP0136 and TP0548 loci can remain stable for years. In addition, the recently determined upper limit for the mutation rate in yaws treponemes [60] combined with the fact that both TPA and TPE are genetically almost identical [61], provides another line of evidence that the above-mentioned allelic profiles are likely to be stable for at least several years of human infection.

In this study, seven samples (6%) belonged to the Nichols-like TPA group and all of them were from northern France, including Paris. The remaining 94% of samples belonged to the SS14-like group of TPA strains. A previous analysis of 2,506 clinical samples, which were classified with respect to the Nichols-like or the SS14-like cluster, revealed 177 (7.1%) clinical samples belonged to the Nichols-like cluster of TPA strains [18, 34, 50, 53]. Data from the present study are therefore in agreement with the meta-analysis of Nichols-like and SS14-like clinical samples. Compared to SS14-like strains/isolates, Nichols-like strains/isolates appear to be more frequently represented by TPA reference strains [34] and appear to be more genetically diverse [5, 19, 21]. Moreover, the proportion of samples clustering with the Nichols-like group differs geographically [4, 34]. As shown in this study, patients infected with Nichols-like TPAwere more often older than 40 years (*p* = 0.041) and more often diagnosed with secondary syphilis (*p* = 0.033) compared to patients infected with SS14-like TPA. Although the biological meaning of this finding remains unknown, it could reflect differences in both the pathophysiology of Nichols-like and SS14-like TPA and/or epidemiological differences in French TPA-infected patients, i.e., belonging to separate subpopulations. Patients belonging to different age groups could possess different behavior patterns and therefore different allelic profiles could circulate in different age groups.

In this study, 102 clinical samples containing TPA (86.4%) harbored the A2058G mutations in both *rrn* operons and none of the examined samples contained the A2059G mutations. Mutations causing macrolide resistance were detected to a similar extent in both SS14-like and Nichols-like clinical samples (88.2% and 71.4%, respectively). This high prevalence of mutations causing macrolide resistance corresponds with the high prevalence of these mutations found in studies from other European countries (reviewed in [35]).

Mutations causing macrolide resistance (A2058G mutations in both *rrn* operons) were found to be associated with allelic profile 1.3.1. This association was also found in previous studies including TPA-containing samples from the Czech Republic (SU2; [20]), and France and Switzerland (1.3.1., [21]. Moreover, the macrolide resistant allelic profile 1.3.1 is a major genotype of the omega wide-spreading cluster identified by Arora and colleagues [5]. All these findings may explain why allelic profile 1.3.1, likely due to the presence of the macrolide resistant A2058G mutation, represents one of the most successful TPA genotypes in several European countries. Although there is no evidence of pathophysiological differences between TPA strains/isolates of different genotypes, the above-mentioned associations support the scenario that different TPA strains could differ in their pathogenicity and/or other parameters including e.g. transmission efficiency.

Among the completely typed samples, four allelic profiles (1.1.1, 1.3.1, 1.1.8, and 9.7.3) were found to contain 23S rRNA genes with both the macrolide susceptible version and macrolide resistant version. As suggested in an earlier work [35], this supports the concept that mutations encoding macrolide resistance have emerged several times in different TPA strains, independently of strain background. This prediction is also supported by the recent emergence of mutations encoding macrolide resistance in yaws treponemes [4, 62].

Findings from this study indicate that the group of TPA strains infecting Europeans is quite genetically diverse and that additional typing studies, in other European and non-European countries, will be needed to reveal the full genetic diversity of TPA pathogens in different geographical areas. Mapping the genetic diversity of TPA strains will provide insights into syphilis epidemiology as well as syphilis evolution.

## Supporting information

S1 Table Primers used for nested-PCR amplification. (DOCX)

S2 Table Allelic variants at the TP0136, TP0548, and TP0705 loci with discovered nucleotide changes and corresponding amino acid changes. (DOCX)

S1 Appendix All allelic variants at the TP0136, TP0548, and TP0705 loci identified in this study and by Grillová et al. 2018. This file includes the (1) allelic variants of TP0136 in SS14-like strains, (2) allelic variants of TP0136 in Nichols-like strains, (3) allelic variants of TP0548 in SS14-like strains, (4) allelic variants of TP0548 in Nichols-like strains and (5) allelic variants of TP0705 (regardless of SS14- and Nichols-like cluster), each on a specific individual sheet.
(XLSX)
