# Supplementary material for: Multi-locus sequence typing of Treponema pallidum subsp. pallidum present in clinical samples from France: Infecting treponemes are genetically diverse and belong to 18 allelic profiles
Source: PLoS One. 2018 Jul 19;13(7):e0201068. doi: 10.1371/journal.pone.0201068 (PMC6053231; doi:10.1371/journal.pone.0201068)
Supplement: S1 Table — (DOCX) [file pone.0201068.s001.docx]

**S1 Table. Primers used for nested-PCR amplification.**

| Locus | External primers (5-3) | Coordinates^1^ | Length of PCR product | Source/reference | Internal primers (5-3) | Coordinates^1^ | Length of PCR product | Source/reference |
| --- | --- | --- | --- | --- | --- | --- | --- | --- |
| TP0136 | AACCCGTTAGCGCCCAACAT | 157804-157823 | 1789 bp | Matějková et al., 2009 | AGTGTCTTCCTCGTCCGTTC | 158206-158225 | 1206 bp | Woznicová et al., 2007 |
|  | TCCCAGCTCAGCCGAATCTC | 159570-159589 |  |  | CACGTGGTGGTGTCAAACTT | 159392-159411 |  |  |
|  |  |  |  |  |  |  |  |  |
| TP0548 | TGGGGCACTAAACCGGAAGA | 593136-593155 | 1567 bp | Matějková et al., 2009 | GCGGTCCCTATGATATCGTGT | 593285-593305 | 1065 bp | Woznicová et al., 2007 |
|  | TACGGGCATTTGCGGATAGG | 594683-594702 |  |  | GAGCCACTTCAGCCCTACTG | 594330-594349 |  |  |
|  |  |  |  |  |  |  |  |  |
| TP0705 | GGTCTATATGCAGCCCTTCTTC | 772663-772684 | 1181 bp | Grillová et al. 2018 | TGCGGCTTATCCTGATGAATAG | 772917-772938 | 803 bp | Grillová et al. 2018 |
|  | GCTTGAGAACGATACCGGATAC | 773822-773843 |  |  | TATTCTGCGGCGTTGGATAG | 773700-773719 |  |  |
|  |  |  |  |  |  |  |  |  |
| 23S rDNA^2^ | CGAAGGGAAGCAGGTGTAGT | 234704-234723, 283149-283168 | 1666 and 1658 bp | Lukehart et al., 2004, Grillová et al. 2018 | GTACCGCAAACCGACACAG | 234768-234786 | 629 bp | Lukehart et al., 2004 |
|  | GCGCGAACACCTCTTTTTAC | 236350-236369 |  |  | AGTCAAACCGCCCACCTAC | 235378-235396 |  |  |
|  | GAACCGTCCCTGAAAACTCA | 284787-284806 |  |  |  |  |  |  |

^1^Based on the Nichols genome (CP004010.2).

^2^Both copies of 23S rDNA gene were amplified.
