# Supplementary material for: Multi-locus sequence typing of Treponema pallidum subsp. pallidum present in clinical samples from France: Infecting treponemes are genetically diverse and belong to 18 allelic profiles
Source: PLoS One. 2018 Jul 19;13(7):e0201068. doi: 10.1371/journal.pone.0201068 (PMC6053231; doi:10.1371/journal.pone.0201068)
Supplement: S2 Table — (DOCX) [file pone.0201068.s002.docx]

**S2 Table. Allelic variants at the TP0136, TP0548, and TP0705 loci with discovered nucleotide changes and corresponding amino acid changes.**

| Allelic variants | Nucleotide change(s) | Amino acid change(s) |
| --- | --- | --- |
| TP0136_1 | SS14 strain identical | - |
| TP0136_2 | SS14, 733 C=>A | 245 H=>N |
| TP0136_3 | Nichols, del 989-994, 1205 G=>A | del 333-334, 402 G=>D |
| TP0136_9 | Nichols, 1205 G=>A | 402 G=>D |
| TP0136_14^1^ | SS14, 1145 C=>T | 382 T=>M |
| TP0548_1 | SS14 strain identical | - |
| TP0548_2 | Nichols, 170 G=>A | 57 G=>D |
| TP0548_3 | SS14, 154 G=>A, 158 G=>A | 52 G=>R, 53G=>E |
| TP0548_7 | Nichols, 170 G=>A, 208 C=>A | 57 G=>D, 70 G=>K |
| TP0548_11 | SS14, 170 G=>A | 57 C=>Y |
| TP0548_17^1^ | SS14, 491 G=>A | 164 S=>N |
| TP0548_18^1^ | SS14, 154 G=>A, 158 G=>A, 635 A=>G | 52 G=>R, 53G=>E, 212 K=>R |
| TP0548_19^1^ | SS14, 154 G=>A, 158 G=>A, 505 G=>A | 52 G=>R, 53G=>E, 169 G=>S |
| TP0548_20^1^ | Nichols, 170 G=>A, 208 C=>A, 500 A=>G | 57 G=>D, 70 G=>K, 168 H=>R |
| TP0548_21^1^ | SS14, 96 G=>T^2^, 154 G=>A, 158 G=>A | 32 no change, 52 G=>R, 53G=>E |
| TP0548_22^1^ | SS14, 300 C=>A | 100 N=>K |
| TP0548_23^1^ | SS14, 127 A=>G, 154 G=>A, 158 G=>A | 43 M=>V, 52 G=>R, 53G=>E |
| TP0705_1 | 1873 A=>G, 2122 G=>A | 625 M=>V, 708 G=>S |
| TP0705_2 | 1459 A=>C, 1873 A=>G, 2122 G=>A | 487 I=>L, I625 M=>V, 708 G=>S |
| TP0705_3 | 1516 G=>A | 506 A=>T |
| TP0705_8 | 1517 C=>T | 506 A=>V |
| TP0705_9 | 2122 G=>A | 708 G=>S |
| TP0705_11^1^ | 1873 A=>G, 1957 G=>T, 2122 G=>A | 625 M=>V, 653 G=>W, 708 G=>S |
| TP0705_12^1^ | 2023 G=>T | 675 A=>T |
| TP0705_13^1^ | 1818 G=>A^2^, 1873 A=>G, 2122 G=>A | 606 no change, 625 M=>V, 708 G=>S |

^1^allelic variant first described in this study

^2^silent mutation
